# Synthesis and Activity Evaluation of 2-(1-naphtho[2,1-*b*]furan-2-yl-carbonyl)-3,5-disubstituted-2,3-dihydro-1H-pyrazoles

**DOI:** 10.4103/0250-474X.49090

**Published:** 2008

**Authors:** M. N. Kumaraswamy, C. Chandrashekhar, H. Shivakumar, D. A. Prathima Mathias, K. M. Mahadevan, V. P. Vaidya

**Affiliations:** Department of PG Studies and Research in Chemistry, School of Chemical Sciences, Kuvempu University, Jnana Sahyadri, Shankaraghatta-577 451, India; 1 Department of PG Studies and Research in Pharmacology, SCS College of Pharmacy, Harapanahalli-583 131, India

**Keywords:** Naphtho[2,1-*b*]furan, naphthofuropyrazoles, pyrazoles, pharmacological activities

## Abstract

Ethyl naphtho[2,1-*b*]furan-2-carboxylate (2) on reaction with hydrazine hydrate in presence of acid catalyst in ethanol medium affords naphtho[2,1-b]furan-2-carbohydrazide (3). The reaction of substituted acetophenones (4a-c) with aromatic aldehydes (5a-e) produces chalcones (6a-o) via the Claisen condensation. The reaction of naphtho[2,1-*b*]furan-2-carbohydrazide (3) with chalcones (6a-6o) in presence of acetic acid as catalyst in dioxane produces 1-(naphtho[2,1-*b*]furan-2-yl-carbonyl)-3,5-disubstituted-2,3-dihydro-1H-pyrazoles (7a-o). The structures of newly synthesized compounds have been established by elemental analysis and spectral studies. The compounds 7a-o have been evaluated for their antimicrobial activity and some selected compounds evaluated for antiinflammatory, analgesic, anthelmintic, diuretic and antipyretic activities.

The pyrazole-based derivatives have shown several biological activities, many of them are currently being tested and/or clinically evaluated for new drug discovery^1^. Various pyrazole and pyrazoline derivatives have been reported to possess antinociceptive effect in mice^2^, antimicrobial^3^,^4^, insecticidal^5^ and local anesthetic^6^ activities. Some of the pyrazole derivatives also serve as intermediates in dye industry^7^ and act as growth inhibitors of phytopathogenic fungi^8^. The biheterocyclic compounds in which pyrazole moiety is coupled with furan or benzofuran nucleus exhibit antimicrobial and antiinflammatory activities^9^–^10^. However there are no reports in literature concerning coupling of pyrazole ring with another biologically active naphtho[2,1-*b*]furan nucleus, either directly or through carbon bridge.

Receiving impetus from these reports, guided by the principle that combination of two or more biologically active heterocyclic systems enhances the biological profile of molecules many folds^11^ and in continuation of our research for more potent derivatives of naphtho[2,1-*b*]furan derivatives^12^–^17^, we report in this paper synthesis and pharmacological investigation of novel biheterocyclics, 1-(naphtho[2,1-*b*]furan-2-yl-carbonyl-3,5-disubstituted-2,3-dihydro-1*H*-pyrazoles (7a-7o) bridged via carbonyl group.

## MATERIALS AND METHODS

Melting points were determined with open capillary and are uncorrected. IR spectra were recorded in KBr pellets by using Shimadzu FT-IR 8000 Spectrometer. ^1^H NMR and ^13^C NMR were recorded in DMSO-d_6_ on Bruker-400 MHz Spectrometer. Chemical shifts are recorded in δ relative to TMS as internal standard. Mass spectral data were obtained on a Brucker Apex-II Mass Spectrometer. Elemental analyses were performed using a Vario-EL elemental analyzer. Purity of the compounds was checked by TLC. All the animals were maintained under standard conditions and had access to pelleted animal feed and water. The study protocols were approved by the institutional animal ethics committee (CPCSEA Regd.No.157/1999).

### Chemical synthesis:

To a solution of 2-hydroxy-1-naphthaldehyde (1) (5.16 g, 0.03 mol) in dry N,N-dimethylformamide (25 ml), ethylchloroacetate (3.66 g, 0.03 mol) and anhydrous potassium carbonate (12.4 g, 0.9 mol) were added and the reaction mixture was refluxed on water bath for 24 h. The reaction mixture was then poured into ice cold water, to obtain the product ethyl naphtho-[2,1-*b*]furan-2-carboxylate (2) as solid, which was collected by filtration, dried and recrystallised from ethanol.

A mixture of ethyl naphtho-[2,1-*b*]furan-2-carboxylate (2) (2.40 g, 0.01 mol), catalytic amount of concentrated hydrochloric acid and hydrazine hydrate (1 g, 0.02 mol) were refluxed in absolute ethanol (25 ml) for 2 h on water bath. Then the reaction mixture was cooled to room temperature, the solid thus obtained was filtered and dried. The product, naphtho-[2,1-*b*]furan-2-carbohydrazide (3) obtained was recrystallised from ethanol.

Freshly distilled acetophenone (4) (2.6 g, 0.0215 mol) was added to a cooled mixture of sodium hydroxide (1.1 g, 0.0275 mol), water (10 ml) and rectified spirit (6 ml). To this mixture 4-methoxybenzaldehyde (5b) (2.9 g, 0.0215 mol) was added drop wise maintaining the temperature at < 20°. After the addition was over, the reaction mixture was stirred vigorously until the reaction mixture became thick. It was cooled overnight in ice chest. The solid obtained was filtered and washed with cold water and rectified spirit. The crude chalcone, 3-(4-methoxyphenyl)-1-phenylprop-2-en-1-one (6b) was dried and recrystallised from rectified spirit. The compounds 6a, 6c-o were synthesized by the same method described above using 4-chloroacetophenone, 4-hydroxyacetophenone and different aromatic aldehydes.

To a solution of 3-(4-methoxyphenyl)-1-phenylprop-2-en-1-one (6b) (1.38 g, 0.005 mol) in dioxane (25 ml), acetic acid (0.5 ml) was added and the mixture was kept for stirring for 30 minutes. To this mixture naphtho-[2,1-*b*]furan-2-carbohydrazide (3) (1.01g, 0.005 mol) was added and refluxed for 24 h. After completion of the reaction, the reaction mixture was poured to ice cold water, solid separated was filtered and dried. The crude product was recrystallised from ethanol. The compounds 7a, 7c-o were synthesized similarly from 6a, 6c-o. The characterization data of the synthesized compounds are reported in Table 1. The structures of newly synthesized compounds were elucidated by IR, NMR spectral studies, which are reported in Table 2.

**TABLE 1:** CHARACTERIZATION DATA OF THE COMPOUNDS 7a-O

Compd.	R_1_	R_2_	Mol. formula	Yield (%)	mp^(0)^	Found (Calcd.) % N
7a	H	H	C_28_H_20_ N_2_O_2_	68	235	6.64 (6.73)
7b	H	4-OCH3	C_29_H_22_ N_2_O_3_	65	241	6.18 (6.27)
7c	H	4-OH	C_28_H_20_ N_2_O_3_	63	248	6.39 (6.48)
7d	H	4-Cl	C_28_H_19_N_2_O_2_Cl	70	>250	6.14 (6.21)
7e	H	4-NO2	C_28_H_19_ N_3_O_4_	62	>250	9.00 (9.11)
7f	4-Cl	H	C_28_H_19_N_2_O_2_Cl	71	>250	6.16 (6.21)
7g	4-Cl	4-OCH3	C_29_H_21_N_2_O_3_Cl	67	>250	5.70 (5.82)
7h	4-Cl	4-OH	C_28_H_19_N_2_O_3_Cl	68	>250	5.91 (6.00)
7i	4-Cl	4-Cl	C_28_H_18_N_2_O_2_Cl_2_	70	>250	5.68 (5.77)
7j	4-Cl	4-NO2	C_28_H_18_N_3_O_4_Cl	65	>250	8.38 (8.47)
7k	4-OH	H	C_28_H_20_N_2_O_3_	66	>250	6.39 (6.48)
7l	4-OH	4-OCH3	C_29_H_22_N_2_O_4_	71	>250	5.99 (6.06)
7m	4-OH	4-OH	C_28_H_20_N_2_O_4_	65	>250	6.13 (6.25)
7n	4-OH	4-Cl	C_28_H_19_N_2_O_3_Cl	68	>250	5.91 (6.00)
7o	4-OH	4-NO2	C_28_H_19_N_3_O_5_	67	>250	8.70. (8.80)

Satisfactory C and H analysis was obtained for all the compounds.

**TABLE 2:** IR AND NMR SPECTRAL DATA OF 2-(1-NAPHTHO[2,1-*b*]FURAN-2-YL-CARBONYL)-3,5-DISUBSTITUTED-2,3- DIHYDRO-1*H*-PYRAZOLES

Comp.	R_1_	R_2_	IR (KBr) (C=O)	1H NMR
7a	H	H	1694	δ 6.1 (s, ^1^H, NH), δ 7.3-8.5 (m, NCHPh +CHCPh +17ArH)
7b	H	4-OCH_3_	1663	δ 3.8 (s, ^3^H, OCH_3_), δ 6.0 (s, ^1^H, NH), d 7.0-8.5 (m, NCHPh+CHCPh +16ArH)
7c	H	4-OH	1675	δ 4.7 (b, ^1^H, OH), δ 6.1 (s, ^1^H, NH), δ 7.3-8.6, (m, NCHPh+CHCPh +16ArH)
7d	H	4-Cl	1683	δ 5.8 (s, ^1^H, NH), δ 7.4-8.6 (m, NCHPh+ CHCPh +16ArH)
7e	H	4-NO_2_	1678	δ 6.2 (s, ^1^H, NH), δ 7.2-8.8 (m, NCHPh+ CHCPh +16ArH)

IR streching frequencies are measured in cm^−1^ and ^1^H NMR chemical shift are expressed in δ ppm, values in comparison with standard TMS.

### Antimicrobial activity:

The *in vitro* antimicrobial activity was carried out against 24 h old cultures of two bacteria and two fungi by cup-plate method^18^. The compounds (7a-o) have been tested for their antibacterial activity against *Pseudomonas aeruginosa* and *Staphylococcus aureus* and antifungal activity against *Aspergillus niger* and *Curvularia lunata*. Chloramphenicol and fluconazole were used as standards for antibacterial and antifungal activity respectively. The compounds were tested at a concentration in of 0.001 mol/ml in DMF against all the organisms. The zone of inhibition was compared with the standard drug after 24 h of incubation at 25° for antibacterial activity and 48 h at 30° for antifungal activity. The results are reported in Table 3.

**TABLE 3:** ANTIMICROBIAL ACTIVITY OF THE COMPOUNDS 7a-o

Compd.	Zone of inhibition in mm			

	*P. aeruginosa*	*S. aureus*	*Aspergillus niger*	*Curvularia lunata*
7a	17	17	17	18
7b	15	16	14	15
7c	15	15	16	17
7d	17	16	15	17
7e	16	16	16	15
7f	17	15	16	16
7g	15	16	16	17
7h	16	17	19	18
7i	19	19	18	18
7j	17	16	18	17
7k	19	18	18	19
7l	20	19	19	18
7m	19	18	19	18
7n	18	20	19	19
7o	19	18	20	19
Standard	24	26	24	22
DMF	+ ve	+ ve	+ ve	+ ve

Zone of inhibition expressed in mm. The Cup-plate method was followed and chloramphenicol and flucanazole were used as standards. The concentration of the drug used is 0.001 mol/ml in DMF

### Antiinflammatory activity:

The antiinflammatory activity was evaluated by a rat paw edema method, which is based on plethysmographic measurement of carrageenan-induced acute rat paw edema^19^–^20^. For this study, wistar rats of either sex, weighing between 100-200 g, were used and were divided into 7 groups, of 4 animals each. The group I served as control, the group II received ibuprofen and served as standard, and the groups III-VII received orally the test compounds. These drugs were administered 1 h before the injection of carrageenan. After 1 h all the animals were injected subcutaneously with a suspension of carrageenan in Tween-80 (0.1%, 0.05 ml) solution to the left hind paw in the subplantar region and the paw volume was measured immediately. After 3 h the paw volume was measured in control, in standard and in test groups. Percent inhibition of paw volume was calculated using the formula, % inhibition= (1-Vt/Vc)×100, where, Vt is the mean increase in the paw volume in test animals group and Vc is the mean increase in the paw volume in control group. The results are reported in Table 4.

**TABLE 4:** ANTIINFLAMMATORY ACTIVITY OF COMPOUNDS 7a-e

Compd.	Group	Paw volume ±SEM after 3 h	% Inhibition of edema after 3 h
Control	I	0.98±0.02	----
Ibuprofen	II	0.20±0.01	79.59
7a	III	0.57±0.02	41.83
7b	IV	0.42±0.01	55.14
7c	V	0.48±0.02	51.02
7d	VI	0.51±0.01	52.04
7e	VII	0.53±0.01	45.91

The control used Tween-80 (0.1%, 1 ml), and standard used ibuprofen and the concentration of drug used 30 mg/kg body weight in Tween-80 (0.1%) solution. Number of animals used in each group is 4

### Analgesic activity:

Analgesic activity was determined by the method based on acetic acid- induced writhing in mice^21^–^22^. For this experiment, colony bred swiss mice of either sex weighing 25-35 g mice were divided into control, standard and different test groups containing 6 animals each. The results are reported in Table 5. Percent inhibition of writhing was calculated using the formula, % Inhibition= (1−Nt/Nc)×100, where, Nt is the mean number of writhing in test animals and Nc represented mean number of writhing in control.

**TABLE 5:** ANALGESIC ACTIVITY OF COMPOUNDS 7a-e

Comp.	Group	R_1_	R_2_	Mean number of writhings ±SEM	% Protection
Control	I	----	----	42.15±3.16	----
Aspirin	II	----	----	12.20±1.50	71.00
7a	III	H	H	18.23±2.18	56.74
7b	IV	H	4-OCH_3_	15.31±1.96	63.67
7c	V	H	4-OH	17.84±2.14	57.67
7d	VI	H	4-Cl	14.78±1.83	64.93
7e	VII	H	4-NO_2_	16.43±2.02	61.07

Acetic acid-induced writhing method, Tween-80 (0.1%, 0.5 ml) used as control, aspirin used as standard, concentration of drug used 100 mg/kg in 0.1% Tween-80 suspension, Number of animals used in each group 6

### Anthelmintic activity:

Anthelmintic activity was evaluated using *Pheritima posthuma* (class-Annelida and order-Oligichaeta). The technique adopted was that described by Giand *et al.*^23^ with slight modification^24^. The worms with normal motility were selected for the experiment and albendazole is used as standard for comparison of the activity. The results are tabulated in Table 6.

**TABLE 6:** ANTHELMINTIC ACTIVITY OF COMPOUNDS 7a-o

Comp.	R_1_	R_2_	Mean paralyzing time (min)	Mean death time (min)
Standard	----	-----	35	44
7a	H	H	71	144
7b	H	4-OCH_3_	100	150
7c	H	4-OH	70	119
7d	H	4-Cl	71	144
7e	H	4-NO_2_	177	281
7f	4-Cl	H	218	259
7g	4-Cl	4-OCH_3_	105	119
7h	4-Cl	4-OH	101	143
7i	4-Cl	4-Cl	106	173
7j	4-Cl	4-NO_2_	113	164
7k	4-OH	H	121	192
7l	4-OH	4-OCH_3_	134	175
7m	4-OH	4-OH	72	113
7n	4-OH	4-Cl	83	125
7o	4-OH	4-NO_2_	96	134

Giand *et al*. method was used for the study. 0.1% Tween-80 prepared in 25 ml of 6% dextrose solution used as control, albendazole used as standard, concentration of drug used is 25 mg in 0.1% Tween-80 in 25 ml of 6% dextrose solution, number of animals in each group 4 worms

### Diuretic activity:

The diuretic activity was evaluated on wistar rats using the method reported by Lipschitz^25^. Rats of either sex, weighing between 100-200 g were divided into 7 groups, each containing 6 animals. Group I served as control, group II served as standard. The groups III-VII received orally the test compounds at the dose of 30 mg/kg body weight in Tween-80 (0.1%, 5 ml). Each group of animals was kept in different metabolic cages provided with a wire mesh at the bottom and a funnel to collect urine. Urine excreted was collected after 5 h and the values are tabulated in Table 7.

**TABLE 7:** RESULTS OF DIURETIC ACTIVITY OF COMPOUNDS 7a-e

Comp.	Group	R_1_	R_2_	Volume of urine collected in ml after 5 h.	T/S (Lipschitz value)
Control	I	---	----	8	0.27
Frusemide	II	---	----	29	1.00
7a	III	H	H	14	0.48
7b	IV	H	4-OCH_3_	15	0.52
7c	V	H	4-OH	16	0.55
7d	VI	H	4-Cl	15	0.52
7e	VII	H	4-NO_2_	19	0.65

Control, concentration of drug used is 30 mg/kg in Tween-80 (0.1%, 5 ml) solution. Number of animals in each group 6

### Antipyretic activity:

The antipyretic activity was carried out on wistar rats as described by the method based on yeast-induced hyperpyrexia method^26^. The rats weighing 150-170 g were selected and divided into 6 groups each having 6 animals. The rectal temperature and its hourly variation were recorded at the beginning of the experiment using a digital Telethermometer. The rats showing rise in rectal temperature of 0.5° or more were distributed in to different group of 6 each and test drugs, Group I received tween–80 as control and group-II received paracetamol as standard drug and groups III-VI received test compounds. The decrease in rectal temperature was noted using telethermometer at 1 h intervals up to 3 h. All values are expressed as mean±SEM. The results are reported in Table 8.

**TABLE 8:** RESULTS OF ANTIPYRETIC ACTIVITY OF COMPOUNDS 7b-e

Comp.	Group	R_1_	R_2_	Mean rectal temperature			
				
				0 h	1 h	2 h	3 h
Control	I	----	----	38.7	38.6±0.16	38.5±0.16	38.5±0.04
Paracetamol	II	----	----	38.4	37.9±0.19	37.8±0.17	37.7±0.17
7b	III	H	4-OCH3	38.2	38.1±0.17	38.1±0.11	38.0±0.09
7c	IV	H	4-OH	37.9	37.8±0.22	37.9±0.18	37.8±0.11
7d	V	H	4-Cl	37.8	37.7±0.11	37.8±0.16	37.7±0.14
7e	VI	H	4-NO2	38.5	38.4±0.12	38.2±0.08	38.1±0.15

Yeast-induced hyperpyrexia method, Tween-80 is used as control, paracetamol is used as standard, concentration of drug used is 100 mg in Tween-80. Number of animals used in each group is 6

## RESULTS AND DISCUSSION

**Scheme 1:**
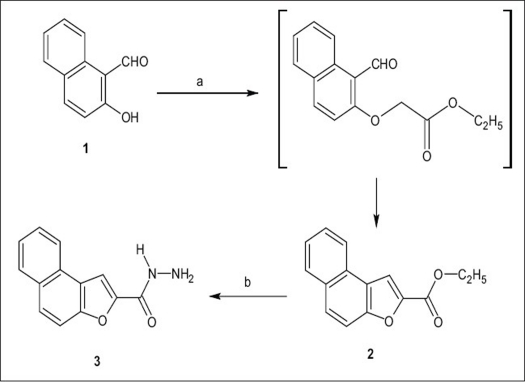
Synthetic route for the synthesis of 3 a-Reaction carried out with ethyl chloroacetate in presence of K_2_CO_3_ and acetone; b- reaction carried out with hydrazine hydrate in ethanol.

The required starting material to accomplish the synthesis of title compounds, ethyl naphtho[2,1-*b*]furan-2-carboxylate (2) was obtained by the reaction of 2-hydroxy-1-naphthaldehyde (1) with ethyl chloroacetate in presence of anhydrous potassium carbonate and in dry DMF at reflux temperature. Both condensation as well as cyclisation occurred in single step and produced ethyl naphtho-[2,1-*b*]furan-2-carboxylate (2) in good yield. The reaction of ethyl naphtho-[2,1-*b*]furan-2-carboxylate (2) with hydrazine hydrate in presence of acid catalyst in ethanol produced naphtho-[2,1-*b*]furan-2-carbohydrazide (3). The synthetic route is shown in Scheme 1.

**Scheme 2:**
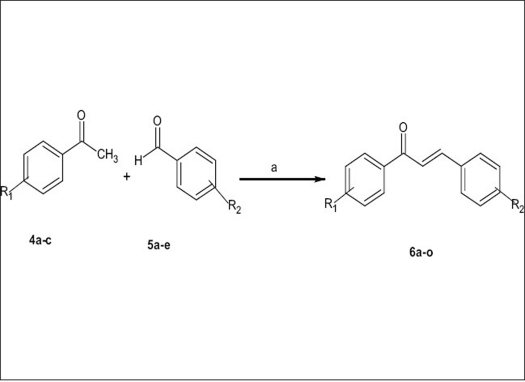
Synthetic route for the synthesis of 6a-o a- Reaction carried out in ethanol solution of sodium hydroxide at 25°

The chalcones (6a-o) were synthesized by Claisen condensation between substituted acetophenones (4a-c) and different aromatic aldehydes^27^ (5a-e). The selection of substituted acetophenones and substituted aromatic aldehydes was based on presence of electron withdrawing and electron releasing groups which would assist in later studies, on structure activity relationship. The synthetic route shown in Scheme 2.

**Scheme 3:**
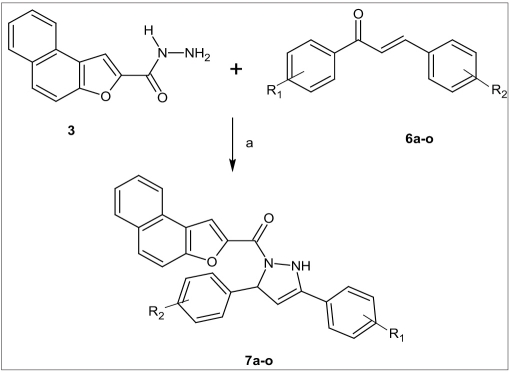
Synthetic route for the synthesis of 7a-o a- Reaction carried out in acetic acid and dioxane at reflux temperature.

The reaction of naphtho[2,1-*b*]furan-2-carbohydrazide (3) with chalcones 6a-o to obtain the title compounds (7a-o) was attempted by employing various reagents and reaction conditions. However, the desired condensation was successful only when the reaction was carried out by using acetic acid as catalyst and dioxane as a solvent at reflux temperature. The target compounds 1-(naphtho[2,1-*b*]furan-2-ylcarbonyl)-3,5-disubstituted-2,3dihydro-1*H*-pyrazoles (7a-o) were obtained in good yield. The synthetic route is shown in Scheme 3.

The compounds containing naphthofuran were known to exhibit a wide spectrum of biological and pharmacological activities^28^–^30^. Hence, it was intrigued to evaluate newly synthesized compounds for antimicrobial, antiinflammatory, analgesic, anthelmintic, diuretic and antipyretic activities by adopting literature procedure.

The newly synthesized compounds were evaluated for antimicrobial activity by cup-plate method. Antibacterial activity was evaluated against *Pseudomonas aerugenosa* and *Staphylococcus aureus* using chloramphenicol as standard drug, The compounds 7i, 7k, 7l, 7m and 7o exhibited activity against *P. aerugenosa*, while compound 7n showed activity against *S. aureus*. For antifungal activity *Aspergillus niger* and *Curvularia lunata* were used as test organisms and fluconazole as standard drug. The compounds 7a-o shows lesser activity than the standard drugs.

Among the compounds tested for antiinflammatory activity, the compounds 7a-e shows lesser activity than the standard drugs. The results of anthelmintic activity indicate that most of the compounds were found to be less active than the standard drugs. The compounds 7a-e were found to display lesser activity when compared to standard drug. The results of antipyretic activity indicated that compound 7e exhibited equal antipyretic activity of reducing the temperature to the extent of 0.4°. Rest of the compounds was less active than the standard drug. From these results it is possible to conclude that most of the synthesized compounds were pharmacologically active. However, no conclusion could be drawn on the effect of electron donating or electron withdrawing groups on pharmacological activities.
